# NLRP3 Deficiency Alleviates Severe Acute Pancreatitis and Pancreatitis-Associated Lung Injury in a Mouse Model

**DOI:** 10.1155/2018/1294951

**Published:** 2018-11-28

**Authors:** Qiang Fu, Zhensheng Zhai, Yuzhu Wang, Lixia Xu, Pengchong Jia, Peng Xia, Chuanjiang Liu, Xu Zhang, Tao Qin, Hongwei Zhang

**Affiliations:** Department of Hepato-Biliary-Pancreatic Surgery, Henan Provincial People's Hospital (People's Hospital of Zhengzhou University), Zhengzhou, Henan 450000, China

## Abstract

The rapid production and release of a large number of inflammatory cytokines can cause excessive local and systemic inflammation in severe acute pancreatitis (SAP) and multiple organ dysfunction syndrome (MODS), especially pancreatitis-associated acute lung injury (P-ALI), which is the main cause of early death in patients with SAP. The NLRP3 inflammasome plays an important role in the maturation of IL-1*β* and the inflammatory cascade. Here, we established a model of SAP using wild-type (NLRP3^+/+^) and NLRP3 knockout (NLRP3^−/−^) mice by intraperitoneal injections of caerulein (Cae) and lipopolysaccharide (LPS). Pathological injury to the pancreas and lungs, the inflammatory response, and neutrophil infiltration were significantly mitigated in NLRP3^−/−^ mice. Furthermore, INF-39, an NLRP3 inflammasome inhibitor, could reduce the severity of SAP and P-ALI in a dose-dependent manner. Our results suggested that SAP and P-ALI were alleviated by NLRP3 deficiency in mice, and thus, reducing NLRP3 expression may mitigate SAP-associated inflammation and P-ALI.

## 1. Introduction

Acute pancreatitis (AP) is an inflammatory disease that is usually diagnosed by acute abdominal pain and an increase in amylase and lipase concentrations in serum. More than 20% of patients with AP have severe acute pancreatitis (SAP). In contrast to mild AP, which has a mortality rate of less than 1%, the mortality rate for SAP is much higher: 10% with sterile and 25% with infected pancreatic necrosis [1]. SAP is frequently associated with acute lung injury and respiratory dysfunction. Several studies have provided some insights into the pathogenesis of acute respiratory insufficiency associated with AP. Nevertheless, the limited knowledge of molecular mechanisms underlying the development of pancreatitis-associated acute lung injury (P-ALI) may explain the lack of specific clinical therapies [2].

Nod-like receptors (NLRs) are intracellular pattern recognition molecules that can detect microbial- and danger-associated molecular patterns. NLRP3, similar to other NLR proteins, plays an important role in forming inflammasomes, which lead to the maturation of interleukin- (IL-) 1 beta (IL-1*β*) [3]. IL-1*β* is closely related to SAP, and its increase is positively correlated with the severity of the disease. The pathological damage to the pancreas, the degree of inflammation, and P-ALI have been shown to be significantly reduced after blocking IL-1*β* expression in SAP [4]. In addition, injured tissues express an array of inflammatory cytokines, including IL-1*β*, tumor necrosis factor alpha (TNF-*α*), and IL-6. The rapid production and release of a large number of inflammatory cytokines can cause excessive local and systemic inflammation and lead to systemic inflammatory response syndrome (SIRS) including vascular leakage, hypovolemia, shock, and multiple organ dysfunction and even cause multiple organ dysfunction syndrome (MODS) [5].

Based on these considerations, the aim of this study was to investigate the role of NLRP3 in SAP and P-ALI. For this purpose, we used two experimental models, NLRP3 knockout (NLRP3^−/−^) mice and NLRP3 inhibitor-treated mice.

## 2. Materials and Methods

### 2.1. Reagents

Caerulein (Cae) and lipopolysaccharide (LPS) were purchased from Sigma-Aldrich (USA). Enzyme-linked immunosorbent assay (ELISA) kits for IL-1*β*, IL-6, and TNF-*α* were purchased from BioLegend. The amylase assay kit was purchased from Jiancheng Biotechnology, Nanjing. Antibodies against NLRP3, myeloperoxidase (MPO) and *β*-actin were purchased from Cell Signaling Technology (CST; USA). TRIzol was purchased from Invitrogen (USA). PrimeScript RT Master Mix and SYBR Premix Ex Taq™ kit were purchased from Takara (Japan). INF-39 was purchased from Selleck Chemicals (USA).

### 2.2. Animal Care

NLRP3^−/−^ C57BL/6 mice were provided by Professor Du Bing, East China Normal University (Shanghai) [6], and wild-type mice (NLRP3^+/+^) were purchased from the Shanghai Laboratory Animal Company. All animal experiments were approved by the Animal Research Ethics Committee of People's Hospital of Zhengzhou University, Zhengzhou, China. All procedures were performed under chloral hydrate anesthesia, and all efforts were made to minimize suffering.

### 2.3. In Vivo SAP Model

The NLRP3^+/+^ and NLRP3^−/−^ mouse models of SAP were established by hourly intraperitoneal injections of Cae for 12 hours (50 *μ*g/kg in 0.2 mL saline), followed by one injection of LPS (10 mg/kg in 0.2 mL saline). In the NLRP3 inhibitor groups, INF-39 was administered at 25 mg/kg or 50 mg/kg 30 min after the first injection of Cae. Twelve hours later, all mice were sacrificed to obtain the pancreas and lungs for subsequent applications. Blood and peritoneal lavage fluid (PLF) were obtained for the detection of IL-1*β*, TNF-*α*, IL-6, and amylase.

### 2.4. HE Staining

The pancreatic and lung tissues were cut into 5-*μ*m sections and stained with hematoxylin and eosin (HE) after fixation. The extent of acinar cell injury and necrosis was quantified by computer-aided morphological examination by experienced morphologists who were blinded to the grouping information. We randomly chose 10 microscopic fields (100×) per tissue sample. The pancreas injury score in pathology is clearly described in Table 1. The severity of lung tissue injury is mainly determined by the degree of neutrophil infiltration and congestion of the alveolar septal capillaries.

### 2.5. ELISA

ELISA was performed to detect the concentrations of IL-1*β*, TNF-*α*, and IL-6 in the serum and PLF according to the instructions of the ELISA kits.

### 2.6. Amylase Detection

Amylase assay kits were used to measure the amylase expression level in serum and PLF according to the manufacturer's instructions.

### 2.7. MPO Immunofluorescence

Tissue sections were deparaffinized, subjected to antigen retrieval and blocked with 5% BSA in PBS for 30 min. The sections were incubated with anti-MPO antibody (10 *μ*g/ml) overnight at 4°C, followed by the appropriate secondary antibody (red, 1:200) at room temperature for 1 hour. Nuclei were stained with DAPI (blue) at a concentration of 1.43 *μ*M. All images were captured by confocal microscopy.

### 2.8. Western Blot Analysis

Total protein from the pancreas and lungs was extracted with RIPA lysis buffer containing 1 mM phenylmethylsulfonyl fluoride (PMSF) according to the manufacturer's instructions (Beyotime Bio, Wuhan, China). Proteins (40 *μ*g) from each sample were loaded and separated using a 12% SDS polyacrylamide gel. The proteins were transferred to a PVDF membrane (Millipore, Bedford, MA, USA), which was then incubated with diluted anti-mouse monoclonal NLRP3 antibody at 1:1000 (CST, USA) and anti-*β*-actin antibody at 1:1000 (CST, USA) at 4°C overnight. The next day, the membranes were incubated with HRP-conjugated secondary antibodies (1:5000) at 37°C for 2 hours, and the signals were visualized with an electrochemiluminescence kit (Pierce, Rockford, IL, USA).

### 2.9. qRT-PCR

Total RNA from the pancreas and lungs was isolated using TRIzol reagent (Invitrogen, USA) and then reverse transcribed into cDNA with PrimeScript RT Master Mix (Takara, Japan) according to the manufacturer's instructions. Quantitative real-time PCR (qRT-PCR) was performed using the SYBR Premix Ex Taq™ kit (Takara, Japan). The expression of NLRP3 and IL-1*β* relative to *β*-actin was determined using the 2-ΔΔCT method. The primers used were NLRP3 (forward) 5′- TTGAAGATGTGGACCTCAAG-3′ and NLRP3 (reverse) 5′- CAATCATGAGTGTGGCTAGA-3′, IL-1*β* (forward) 5′-CACAGAGGATACCACTCCCAACA-3′ and IL-1*β* (reverse) 5′- TCCACGATTTCCCAGAGAACA-3′, *β*-actin (forward) 5′- GTACGCCAACACAGTGCTG-3′ and *β*-actin (reverse) 5′- CGTCATACTCCTGCTTGCTG-3′.

### 2.10. Statistical Analysis

The results are shown as the mean ± SD from at least three separate experiments. Statistical analysis was performed using SPSS 22.0 software, and comparisons were made using Student's t-test and one-way ANOVA. P < 0.05 was considered statistically significant.

## 3. Results

### 3.1. NLRP3 Deficiency Reduced the Severity of SAP

**Figure 1:**
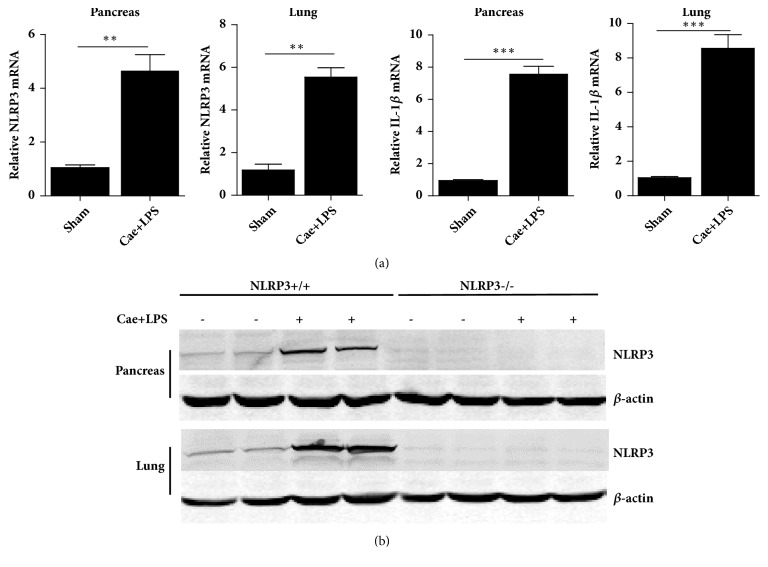
Expression of NLRP3 and IL-1*β* in NLRP3^+/+^ and NLRP3^−/−^ SAP mice model. (a) The NLRP3 and IL-1*β* mRNA level of NLRP3^+/+^ pancreas and lungs tested by q-PCR. (b) NLRP3 protein expression of pancreas and lungs tested by western blotting. *∗∗*P<0.01; *∗∗∗*P<0.001 versus Sham group.

**Figure 2:**
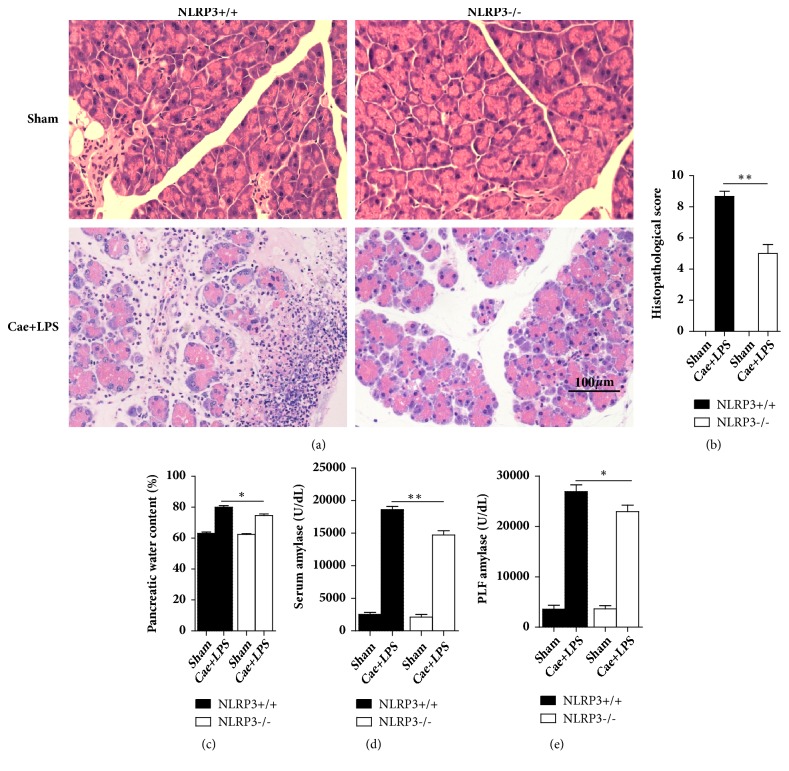
NLRP3 deficiency decreased pancreas injury. (a) The typical HE stain of pancreas. (b) The histopathological score of pancreas. (c) Pancreatic water content percentage. (d) The amylase level of serum. (e) The amylase level of PFL. *∗*P<0.05; *∗∗*P<0.01 versus NLRP3^+/+^ group.

The severity of SAP was significantly alleviated in NLRP3^−/−^ mice compared with that in NLRP3^+/+^ mice. As shown in Figure 1, NLRP3 and IL-1*β* levels were clearly elevated in the pancreas and lungs when the SAP model was established in the NLRP3^+/+^ mice, while the NLRP3^−/−^ mice expressed no NLRP3 protein. HE staining of the pancreatic tissues of NLRP3^+/+^ mice indicated edema, inflammatory cell infiltration, necrosis, and hemorrhage. However, NLRP3^−/−^ mice showed reduced edema in the pancreas, decreased inflammatory cell infiltration, and less tissue necrosis and hemorrhage. The histological scores were significantly lower in the NLRP3^−/−^ mice than in the NLRP3^+/+^ mice, and the tissue water content was also significantly reduced in the former group. The levels of amylase in both serum and PLF decreased in NLRP3^−/−^ mice compared with those in NLRP3^+/+^ mice after the establishment of SAP (Figure 2).

### 3.2. NLRP3 Deficiency Reduced the Levels of IL-1*β*, TNF-*α*, and IL-6 in Serum and PLF

**Figure 3:**
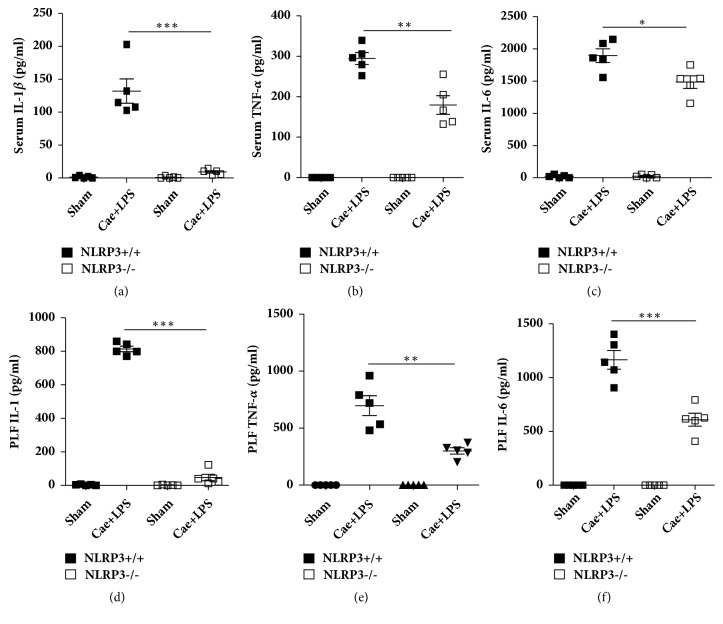
NLRP3 deficiency reduced the levels of IL-1*β* and TNF-*α* in serum and PLF. (a) ELISA-detected protein levels of Serum IL-1*β*. (b) ELISA-detected protein levels of Serum TNF-*α*. (c) ELISA-detected protein levels of Serum IL-6. (d) ELISA-detected protein levels of PLF IL-1*β*. (e) ELISA-detected protein levels of PLF TNF-*α*. (f) ELISA-detected protein levels of PLF IL-6. *∗*P<0.05, *∗∗*P<0.01, and *∗∗∗*P<0.001 versus NLRP3^+/+^ group.

Inflammatory cytokines, such as IL-1*β*, TNF-*α*, and IL-6, are markers of systemic inflammatory responses in SAP. As shown in Figure 3, the levels of IL-1*β*, TNF-*α*, and IL-6 detected by ELISA in serum and PLF were significantly reduced in NLRP3^−/−^ mice compared with those in NLRP3^+/+^ mice.

### 3.3. NLRP3 Deficiency Reduced the Severity of P-ALI

**Figure 4:**
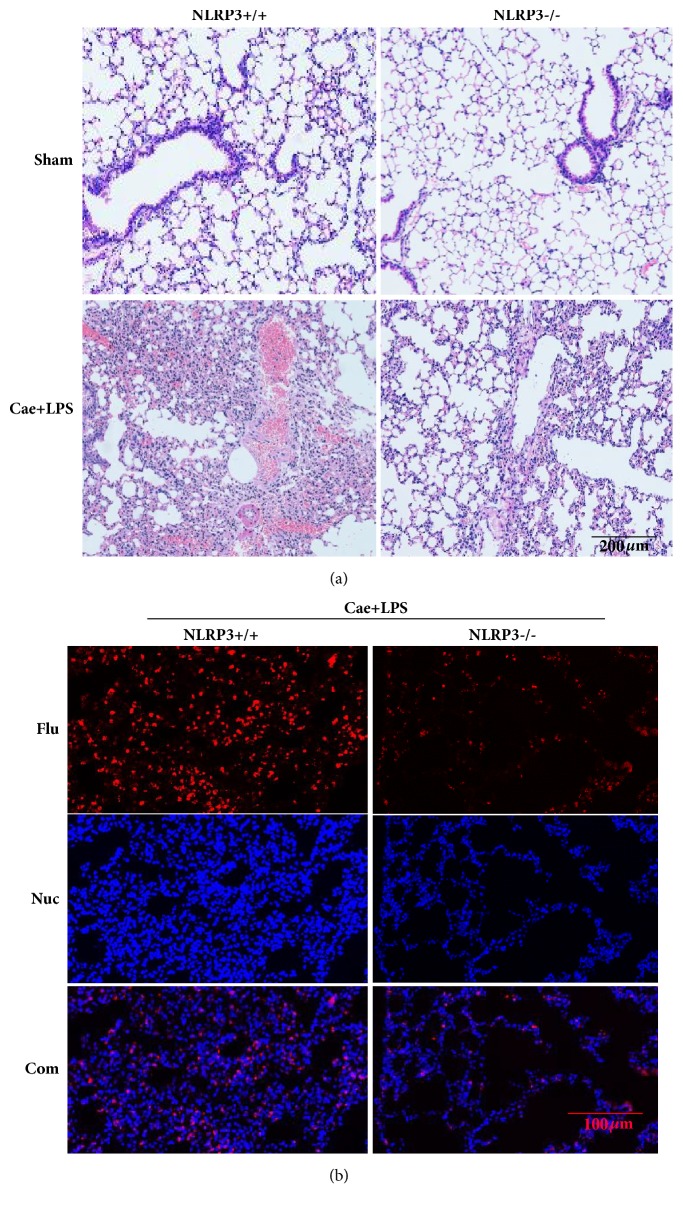
NLRP3 deficiency reduced the injury of lung tissue. (a) Typical HE staining of lung tissue. (b) Typical immunofluorescence of lung tissue.

Lung injury is marked by massive neutrophil infiltration and congestion of the alveolar septal capillaries. As shown in Figure 4, these histological features of lung injury were obvious in NLRP3^+/+^ mice but significantly reduced in NLRP3^−/−^ mice. MPO expression is considered a quantitative marker of neutrophil infiltration. We detected the expression of MPO in lung tissues by immunofluorescence. The results showed that NLRP3 deficiency reduced MPO expression levels in lung tissues.

### 3.4. INF-39 Treatment Reduced the Severity of SAP

**Figure 5:**
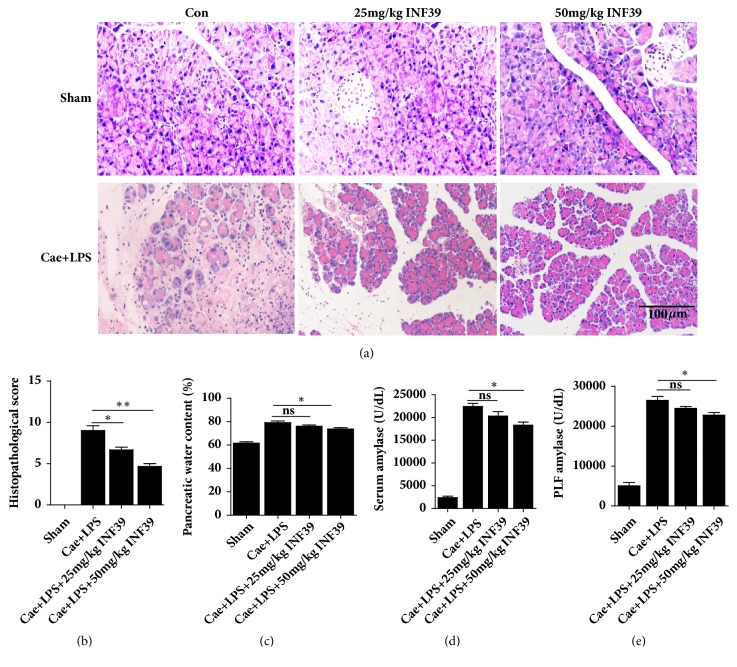
INF-39 treatment reduced the severity of pancreas injury. (a) The typical HE stain of pancreas. (b) The histopathological score of pancreas. (c) Pancreatic water content percentage. (d) The amylase level of serum. (e) The amylase level of PFL.  ^ns^*P*>0.05, *∗P*<0.05, and *∗∗P*<0.01 versus Cae+LPS group.

INF-39, an NLRP3 inhibitor, attenuated damage to pancreatic tissues in the SAP models induced by Cae + LPS in a dose-dependent manner. The typical histological changes were significantly reduced in the Cae + LPS + INF-39 groups compared with those in the Cae + LPS group and were dependent on the dose of INF-39 (Figures 5(a) and 5(b)). In addition, the water content and serum and PLF amylase levels in the Cae + LPS + 50 mg/kg INF-39 group were significantly lower than those in the Cae + LPS group (Figures 5(c), 5(d), and 5(e)).

### 3.5. INF-39 Treatment Reduced the Levels of IL-1*β*, TNF-*α*, and IL-6 in Serum and PLF

**Figure 6:**
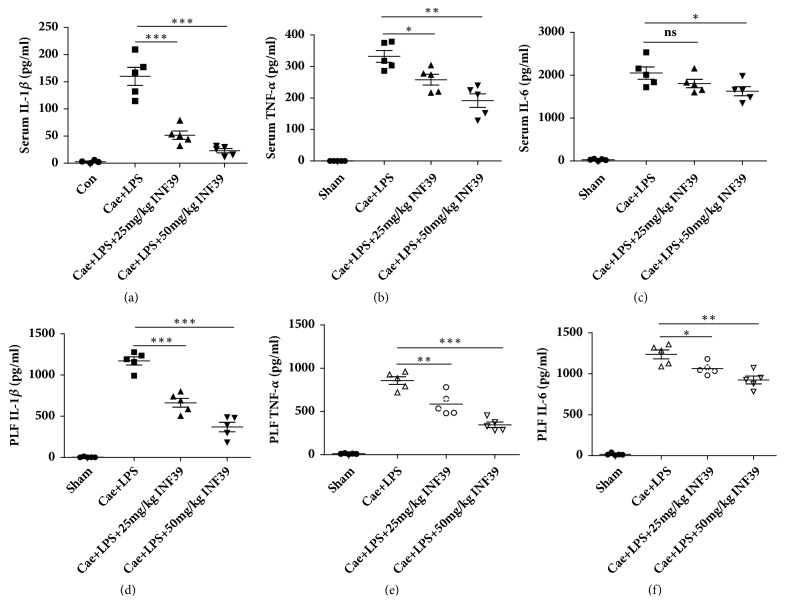
INF-39 treatment reduced the levels of IL-1*β*, TNF-*α*, and IL-6 in serum and PLF. (a) ELISA-detected protein levels of Serum IL-1*β*. (b) ELISA-detected protein levels of Serum TNF-*α*. (c) ELISA-detected protein levels of Serum IL-6. (d) ELISA-detected protein levels of PLF IL-1*β*. (e) ELISA-detected protein levels of PLF TNF-*α*. (f) ELISA-detected protein levels of PLF IL-6.  ^ns^P>0.05, *∗*P<0.05, *∗∗*P<0.01, and *∗∗∗*P<0.001 versus Cae+LPS group.

As described above, IL-1*β*, TNF-*α*, and IL-6 are markers of systemic inflammatory responses after the establishment of SAP. As shown in Figure 6, INF-39 treatment alleviated the serum levels of IL-1*β*, TNF-*α*, and IL-6 in a dose-dependent manner. Similar results were also obtained in PLF after SAP.

### 3.6. INF-39 Treatment Reduced the Severity of P-ALI

**Figure 7:**
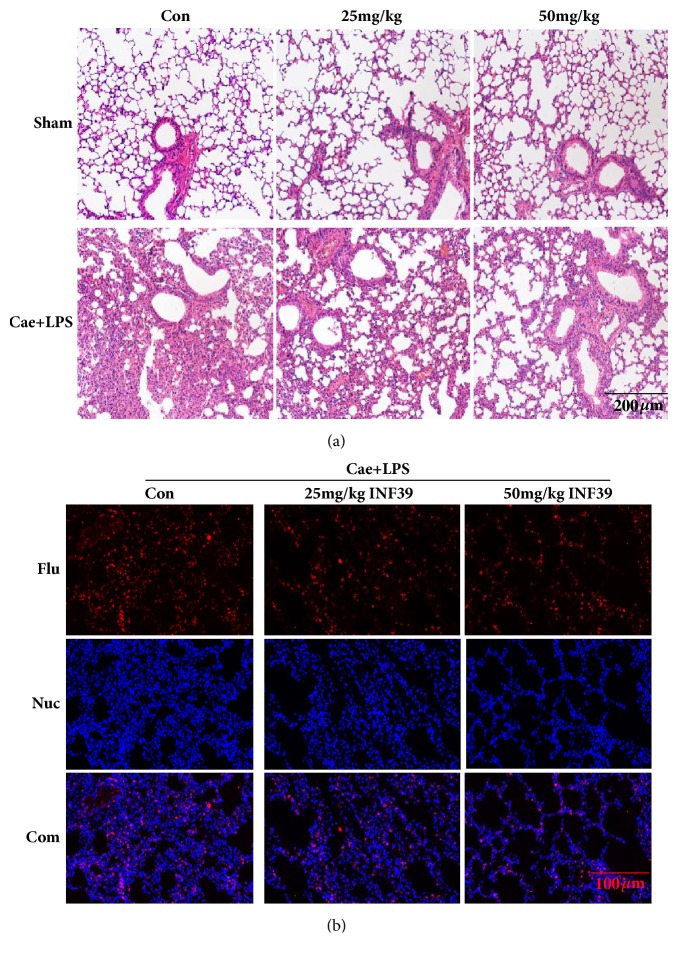
INF-39 treatment reduced the severity of lung injury. (a) Typical HE staining of lung tissue. (b) Typical immunofluorescence of lung tissue.

INF-39 treatment also alleviated P-ALI in the SAP mouse model induced by Cae and LPS. As shown in Figure 7, INF-39 reduced neutrophil infiltration and congestion of the alveolar septal capillaries in a dose-dependent manner. Furthermore, MPO expression levels in the Cae + LPS + 25 mg/kg INF-39 and Cae + LPS + 50 mg/kg INF-39 groups were markedly lower than those in the Cae + LPS group.

## 4. Discussion

The NLRP3 inflammasome is closely related to various diseases, such as chronic inflammation, senility, autoinflammatory syndrome, metabolic diseases, and atherosclerosis and leads to excessive release of inflammatory factors [7–10]. In this study, we established a mouse model of SAP using Cae and LPS and found that the expression of NLRP3 and IL-1*β* in the pancreas and lung tissues of wild-type mice was significantly increased. Our results showed that NLRP3 deletion attenuated the severity of SAP. Histopathological changes, water content of pancreatic tissues, and amylase levels in serum and PLF were significantly reduced in NLRP3^−/−^ mice, consistent with the results of other studies [11]. The NLRP3 inflammasome plays an important role in the progression of AP. The negative regulation of NLRP3 can reduce the degree of pancreatitis [12, 13]. INF-39 is a nontoxic, irreversible NLRP3 inhibitor that blocks NLRP3 activation by irreversibly and directly interacting with NLRP3 and partially inhibiting LPS-driven proinflammatory gene expression [14]. Here, INF-39 reduced the pathological injury to the pancreas and decreased amylase activation in the mouse models of SAP in a dose-dependent manner. Therefore, directly targeting NLRP3 might be an effective treatment for SAP.

The activity of the NLRP3 inflammasome in SAP determines the level of IL-1*β*, which is positively correlated with the severity of SAP [13]. Cytokines play an important role during the pathogenesis and development of SAP [15]. Our results showed that IL-1*β*, TNF-*α*, and IL-6 levels were significantly increased in the serum and PLF in the NLRP3^+/+^ mice with SAP. Interactions among proinflammatory cytokines, among anti-inflammatory cytokines, and between proinflammatory cytokines and anti-inflammatory cytokines, influence the inflammatory process. In particular, proinflammatory factors, once generated and activated, continuously activate and amplify themselves and other cytokines, resulting in an ever-increasing cascade, thereby accelerating the progression of inflammation [16]. The rapid production and release of a large number of inflammatory cytokines can cause excessive local and systemic inflammation in SAP. Our results demonstrated that NLRP3 deletion and INF-39 treatment could almost completely inhibit the maturation and release of IL-1*β* and could block the inflammatory cascades mediated by TNF-*α* and IL-6 in SAP. Therefore, the NLRP3 inflammasome plays an important role in the progression of AP and in the pathogenesis and development of SAP [17].

Activated pancreatic enzymes, inflammatory mediators, and cytokines can cause pulmonary inflammatory responses and the accumulation and activation of neutrophils in lung tissues and, in turn, ALI [18]. The progression from ALI to acute respiratory distress syndrome (ARDS) is a continuous pathological process. Severe ALI is diagnosed as ARDS. The condition is dangerous, and the prognosis is poor [19, 20]. The activation of the NLRP3 inflammasome has also been suggested to be involved in acute lung inflammation after viral/bacterial infection and during the progression of several chronic pulmonary diseases, including idiopathic pulmonary fibrosis, chronic obstructive pulmonary disease, and asthma [21, 22]. However, there are no studies on the function of NLRP3 in the development of P-ALI in SAP.

In the present study, the degree of lung injury and inflammatory cell infiltration was significantly reduced in NLRP3^−/−^ mice. Inhibition of the NLRP3 inflammasome could reduce the severity of P-ALI. Immunofluorescence results showed that the expression of MPO in the lungs was also significantly reduced. MPO, which belongs to the heme-containing proteinase, is produced by neutrophils, monocytes, and macrophages [23, 24]. The increase in MPO in the lungs is an important marker for the accumulation of neutrophils and an important cause of lung injury. Based on our results, NLRP3 deficiency decreased the expression level of MPO in the lungs.

Therefore, we conclude that the deficiency of the NLRP3 inflammasome reduces the severity of SAP and P-ALI. Furthermore, targeted inhibition of the NLRP3 inflammasome with NLRP3 inhibitors may have a protective effect against injury to the pancreas and lungs and reduce the inflammatory cascade and neutrophil infiltration.

## Figures and Tables

**Table 1:** Pancreas pathological scoring criteria.

Score	Edema	Hemorrhage	Inflammatory cell infiltration	Necrosis
0	No	No	0-1/HP	No
1	Mild leaf gap widening	Yes	2-10/HP	Necrotic area
				1%-10%
2	Severe leaf gap widening		11-20/HP	Necrotic area
				11%-20%
3	Acinar gap widening		21-30/HP	Necrotic area
				21%-30%
4	Cell gap widening		≥30/HP or Micro abscess	Necrotic area
				>30%

## Data Availability

No data were used to support this study.
